# The Japanese orthopaedic association at one hundred: a century of Japanese orthopaedics

**DOI:** 10.1007/s00264-026-06994-x

**Published:** 2026-08-12

**Authors:** Yasuharu Nakashima, Hirotaka Kawano

**Affiliations:** 1 Department of Orthopaedic Surgery, Kyushu University, Fukuoka, Japan; 2 The Japanese Orthopaedic Association, Tokyo, Japan; 3 Department of Orthopaedic Surgery, Teikyo University, Tokyo, Japan

**Keywords:** Japanese orthopaedic association, History, Orthopaedics, Centennial, Musculoskeletal health

## Abstract

**Background:**

The Japanese Orthopaedic Association (JOA), founded in 1926, celebrates its centennial in 2026 as one of the world’s largest national orthopaedic societies.

**Methods:**

This article reviews the historical development of the JOA during its first century and highlights major scientific, clinical, educational, and public health achievements of Japanese orthopaedics.

**Results:**

The JOA has played a central role in the development of Japanese orthopaedics through specialist education, clinical practice guidelines, nationwide registries, and the promotion of basic and clinical research. Japanese orthopaedic surgeons have contributed to modern orthopaedics through pioneering surgical techniques, technological innovations, and international academic exchange. These developments have expanded the role of orthopaedics beyond the treatment of musculoskeletal disorders to encompass broader public health challenges associated with population aging and the maintenance of mobility and quality of life.

**Conclusions:**

Over the past century, Japanese orthopaedics has evolved from a small academic discipline into an internationally recognized field, alongside profound demographic and epidemiological changes, particularly the emergence of a super-aged society.

## Introduction

**Fig. 1:**
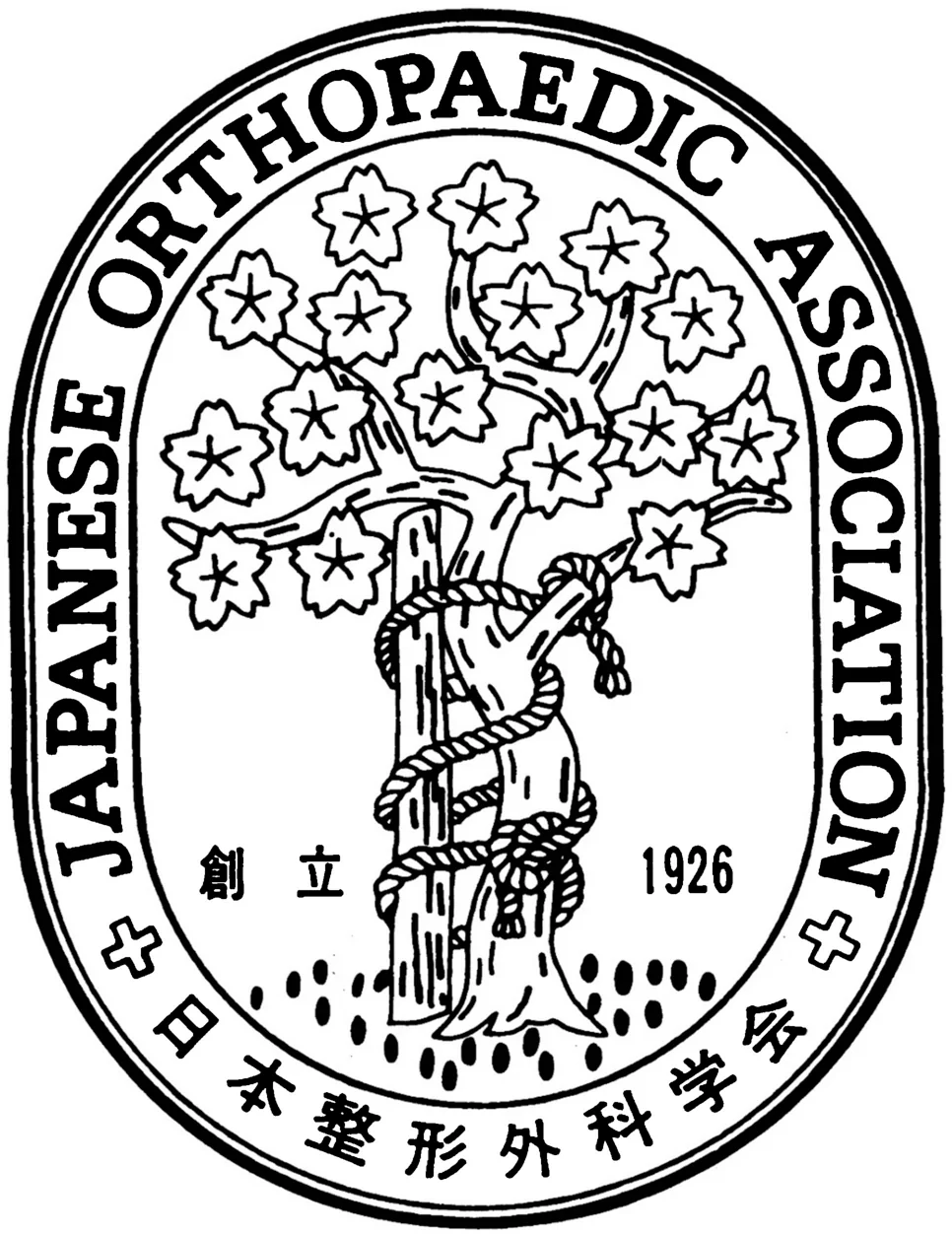
JOA Logo. In 1972, a call for logo designs was issued to members, and this logo was selected. This excerpt is reproduced with permission from the Japanese Orthopaedic Association’s 100-year history book, "The 100-Year History of The Japanese Orthopaedic Association"

The year 2026 marks the 100th anniversary of the Japanese Orthopaedic Association (JOA) [1] (Fig. 1). During the past century, orthopaedic surgery has undergone remarkable advances worldwide, driven by scientific discovery, technological innovation, and changing societal needs. In Japan, these developments have occurred against the unique background of rapid economic growth, universal health insurance, and one of the fastest demographic transitions toward a super-aged society.

Today, the JOA is among the largest orthopaedic societies in the world, representing more than 27,000 members and encompassing every subspecialty of musculoskeletal medicine. Beyond supporting academic activities, the Association has established specialist training systems, developed evidence-based clinical practice guidelines, promoted nationwide registries, and contributed to public awareness of musculoskeletal health. At the same time, Japanese orthopaedic surgeons have introduced numerous innovations that have influenced orthopaedic practice worldwide.

This article reviews the historical evolution of the JOA, emphasizing not only the growth of the Association itself but also the scientific achievements of Japanese orthopaedics, its expanding international engagement, and its contributions to improving musculoskeletal health in Japan and beyond. Many of these achievements are presented in greater detail in the accompanying articles of this commemorative issue.

## The growth of the JOA

The JOA was founded in 1926 under the leadership of Professor Yoshinori Tashiro and other pioneers of Japanese orthopaedics [1]. Among national orthopaedic societies worldwide, it was the ninth to be founded. Academic departments of orthopaedic surgery had already been established at the University of Tokyo and Kyoto University in 1906, followed by Kyushu University in 1909. Approximately two decades of educational and academic development laid the foundation for the establishment of a national professional society.

**Fig. 2:**
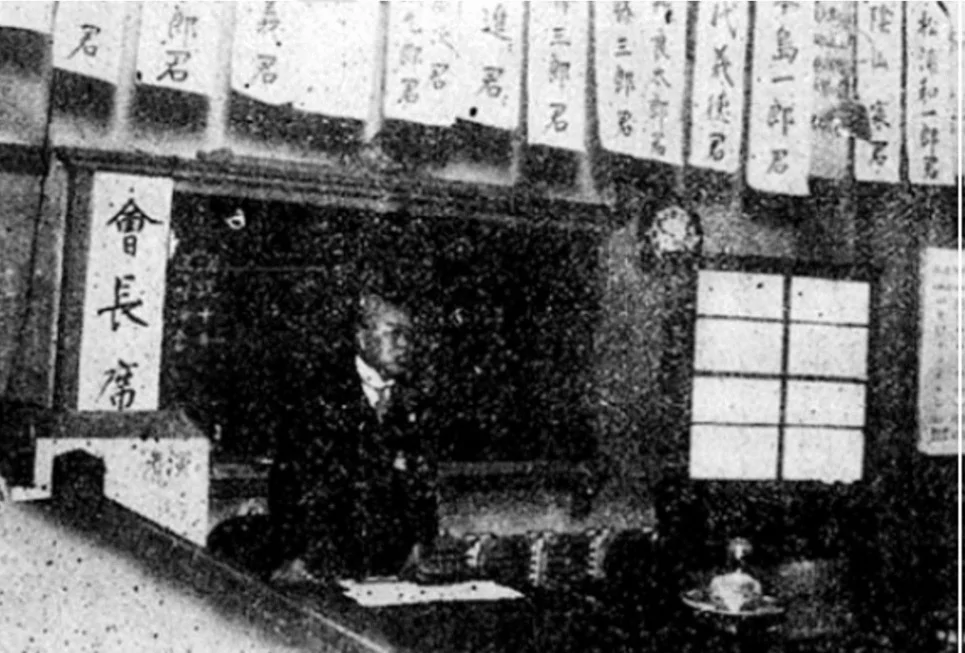
The inaugural scientific meeting of the JOA. Opening remarks by President Yoshinori Tashiro at Tokyo Imperial University in April 3, 1926. The clock is showing 9:25 a.m. Sheets of paper listing the titles of research presentations and the presenters’ names are hung at the front of the hall. This excerpt is reproduced with permission from the Japanese Orthopaedic Association’s 100-year history book, "The 100-Year History of The Japanese Orthopaedic Association"

The inaugural scientific meeting was held in Tokyo with 118 founding members, establishing orthopaedic surgery as an independent academic discipline in Japan (Fig. 2). During its early years, the Association focused primarily on congenital disorders, musculoskeletal infections, tuberculosis, poliomyelitis, and trauma, reflecting the major orthopaedic challenges of the time.

The activities of the Association were inevitably disrupted during the Second World War. Following Japan's post-war reconstruction, however, orthopaedic surgery expanded rapidly in parallel with economic development and the introduction of universal health insurance in 1961. Improved access to medical care, together with advances in diagnostic imaging, biomaterials, and surgical techniques, transformed orthopaedic practice. As life expectancy increased, degenerative diseases of the musculoskeletal system gradually replaced as the major causes of disability, creating new clinical and research priorities.

The growth of the JOA mirrored these societal changes. Membership expanded steadily, and the Association evolved into a comprehensive academic organization supporting education, research, clinical practice, and professional development. A series of subspecialty societies developed under its umbrella, reflecting increasing specialization within orthopaedics while maintaining close academic ties through the JOA.

Education has been one of the Association's principal missions. The JOA has continuously refined its specialist certification system, postgraduate training programs, and continuing medical education to ensure high standards of orthopaedic practice. The publication of evidence-based clinical practice guidelines has further promoted standardized, high-quality patient care throughout Japan.

Over the past century, the annual meeting of the JOA has grown into one of the largest orthopaedic congresses in Asia, serving as a forum for scientific exchange among clinicians, researchers, engineers, and allied health professionals. Through these activities, the Association has contributed not only to the advancement of orthopaedic science in Japan but also to its increasing visibility within the international orthopaedic community.

## Scientific development of Japanese orthopaedics

The scientific development of Japanese orthopaedics has been driven not only by advances in medical science but also by the unique clinical demands of Japanese society. Throughout the past century, Japanese orthopaedic surgeons have translated clinical challenges into innovative surgical techniques, implant technologies, academic initiatives, and engineering solutions that have influenced orthopaedic practice worldwide.

During the 1950 s, Masaki Watanabe transformed arthroscopy from an experimental diagnostic procedure into a practical surgical technique through the development of modern arthroscopes and dedicated surgical instruments [2]. His innovations revolutionized minimally invasive joint surgery and established arthroscopy as an indispensable procedure worldwide.

Building upon the concept of hip preservation, Japanese surgeons introduced several innovative reconstructive procedures for young adults with hip disorders. In 1956, Atsushi Nishio introduced transposition osteotomy of the acetabulum (TOA) for the treatment of developmental dysplasia of the hip (DDH) [3]. This pioneering joint-preserving procedure established the concept of reconstructing acetabular coverage while preserving the native hip joint and laid the foundation for subsequent developments in hip preservation surgery. Rotational acetabular osteotomy (RAO), developed by Tagawa and Ninomiya, expanded the surgical indications for symptomatic acetabular dysplasia and demonstrated favorable long-term outcomes [4]. Yoichi Sugioka developed transtrochanteric rotational osteotomy of the femoral head (TRO) for osteonecrosis of the femoral head, providing a biological alternative to total hip arthroplasty in appropriately selected patients [5]. Together with TOA and RAO, these procedures have become internationally recognized landmark contributions of Japanese orthopaedics to hip preservation surgery.

Japanese spine surgeons also introduced procedures that have had a lasting international impact. In the late 1970 s and 1980 s, several kinds of cervical laminoplasties were developed, providing effective decompression for cervical myelopathy while preserving posterior spinal stability [6]. In the 1990 s, Katsuro Tomita established the concept of total en bloc spondylectomy, creating a new oncological strategy for achieving complete resection of selected primary or metastatic spinal tumours [7]. These procedures remain important components of contemporary spine surgery.

Beginning in the 1990 s, Japanese orthopaedic surgeons also became leaders in computer-assisted orthopaedic surgery. Advances in computer navigation, three-dimensional preoperative planning, image-matching techniques, and robotic-assisted surgery have improved surgical precision in joint replacement, osteotomy, and spinal surgery. These developments were achieved through close collaboration among orthopaedic surgeons, engineers, and industry, representing one of the distinctive characteristics of Japanese orthopaedic research [8].

The JOA has also played an essential role in fostering orthopaedic research. To strengthen translational science, the JOA Research Meeting was established in 1986, providing opportunities for interaction between basic research and clinical medicine. This was followed by the inauguration of the JOA musculoskeletal Tumor Meeting in 1989, which created a national forum dedicated to musculoskeletal oncology. Together with the activities of the JOA Bone and Soft Tissue Tumor Committee, these initiatives promoted nationwide collaborative research and contributed to the standardization of diagnosis, treatment, and clinical research in musculoskeletal tumors.

**Fig. 3:**
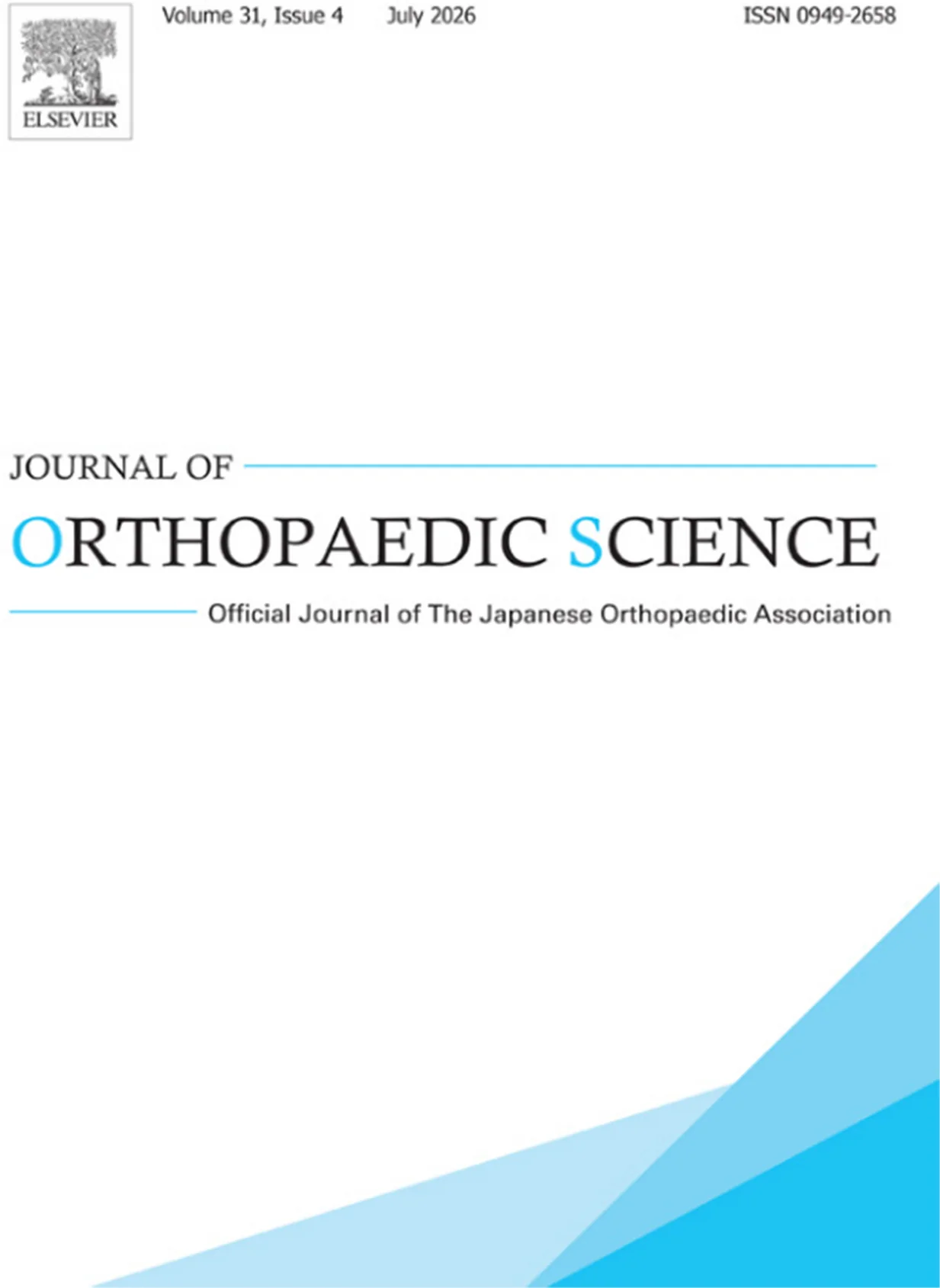
Launch of journal of orthopaedic science in 1996

Another important milestone was the launch of the Journal of Orthopaedic Science in 1996 as the official English-language journal of the JOA (Fig. 3). The journal has provided an international platform for disseminating scientific achievements from Japan and has strengthened academic exchange with orthopaedic communities worldwide.

A major recent achievement has been the establishment of the Japanese Orthopaedic Association National Registry (JOANR) in 2020 [9]. As one of the world's largest nationwide orthopaedic registries, JOANR collects comprehensive data on orthopaedic surgical procedures throughout Japan and serves as a valuable resource for epidemiological research, quality improvement, post-market surveillance of medical devices, and health policy. The registry is also expected to facilitate international collaborative research and accelerate data-driven innovation in musculoskeletal medicine.

Many of the achievements highlighted above are discussed in greater detail in the accompanying articles of this commemorative issue. Collectively, they illustrate a distinctive feature of Japanese orthopaedics: the close integration of clinical practice, engineering, biomaterials science, and basic research, enabling innovations developed in Japan to make enduring contributions to orthopaedic surgery worldwide.

## International exchange

Since the latter half of the twentieth century, JOA has actively promoted international collaboration in education, research, and clinical practice. Long-standing academic relationships have been established with major orthopaedic societies worldwide [10]. Exchange programs have been maintained with the American Academy of Orthopaedic Surgeons (AAOS) and the American Orthopaedic Association (AOA) [10], while close academic ties have also been developed with orthopaedic societies in France (SOFCOT) Germany (DGOU), South Korea (KOA), Taiwan (TOA), Asia–pacific regions (APOA) through reciprocal visits, combined symposia, fellowship programs, and collaborative research. These partnerships have facilitated the exchange of scientific knowledge and contributed to the internationalization of Japanese orthopaedics [11].

Among these international activities, the relationship between the JOA and the Société Internationale de Chirurgie Orthopédique et de Traumatologie (SICOT) has been particularly significant. Japanese orthopaedic surgeons began participating in SICOT congresses in the early post-war period. A particularly significant milestone in the international history of the JOA was the 14th Annual Meeting of SICOT held in Kyoto in 1978. This was the first SICOT Congress convened in Asia and represented a landmark achievement for Japanese orthopaedics. Distinguished Japanese leaders, including Professors Tamikazu Amako and Naokazu Tsuyama played pivotal roles in bringing the Congress to Japan and strengthening the country's international presence. Another milestone was the election of Professor Takao Yamamuro as SICOT President (1993–1996), the first Japanese surgeon to hold this position, reflecting the growing international recognition of Japanese orthopaedics [12]. The close relationship between the JOA and SICOT culminates in the 46th SICOT Orthopaedic World Congress, held in Kyoto in 2026 during the centennial year of the JOA, symbolizing Japan's continuing leadership within the global orthopaedic community.

## Contributions to public health

Beyond scientific research and clinical innovation, the JOA has made substantial contributions to public health through disease prevention, education, and the promotion of healthy longevity.

As Japan became the world's first super-aged society, musculoskeletal disorders emerged as one of the leading causes of disability and loss of independence. In response, the JOA expanded its mission beyond the treatment of individual diseases to the prevention of mobility impairment at the population level.

A landmark achievement was the introduction of the concept of locomotive syndrome in 2007 [13]. Proposed by Professor Nakamura and the JOA, locomotive syndrome describes a condition in which impairment of the musculoskeletal system increases the risk of requiring nursing care. The concept shifted public attention from individual orthopaedic diseases to the preservation of mobility throughout life and became the foundation for nationwide campaigns promoting exercise, early detection, and musculoskeletal health. It has also attracted growing international interest as many countries face similar demographic changes associated with population aging [14].

Public education has also become an important mission of the Association. National awareness campaigns, including Bone and Joint Day, community outreach activities, and educational programs have encouraged early recognition of musculoskeletal disorders and emphasized the importance of maintaining lifelong mobility.

By integrating scientific evidence, clinical practice, professional education, and public awareness, the JOA has contributed to a paradigm shift in musculoskeletal care—from treating disease to preserving mobility and extending healthy life expectancy. This public health perspective is expected to become increasingly important as population aging accelerates worldwide.

## Future perspectives and conclusions

As the JOA enters its second century, it remains committed to advancing orthopaedic science through innovation, education, and international collaboration. Emerging technologies, including artificial intelligence, robotics, regenerative medicine, and nationwide clinical registries, will further transform musculoskeletal care and provide new opportunities for research and evidence-based practice.

Japan's experience as the world's first super-aged society has also demonstrated the importance of preserving mobility to support healthy longevity. The knowledge and experience accumulated over the past century position the JOA to contribute not only to orthopaedic surgery but also to global public health.

The first century of the JOA has been characterized by scientific innovation, international engagement, and a commitment to improving musculoskeletal health. Building on this foundation, the Association is well prepared to contribute to the continued advancement of orthopaedic surgery and musculoskeletal health in the century ahead.

## Data Availability

No datasets were generated or analysed during the current study.
